# Phg1/TM9 Proteins Control Intracellular Killing of Bacteria by Determining Cellular Levels of the Kil1 Sulfotransferase in *Dictyostelium*

**DOI:** 10.1371/journal.pone.0053259

**Published:** 2013-01-02

**Authors:** Marion Le Coadic, Romain Froquet, Wanessa C. Lima, Marco Dias, Anna Marchetti, Pierre Cosson

**Affiliations:** Department of Cell Physiology and Metabolism, Geneva Faculty of Medicine, Centre Médical Universitaire, Geneva, Switzerland; Université de Genève, Switzerland

## Abstract

*Dictyostelium discoideum* has largely been used to study phagocytosis and intracellular killing of bacteria. Previous studies have shown that Phg1A, Kil1 and Kil2 proteins are necessary for efficient intracellular killing of *Klebsiella* bacteria. Here we show that in *phg1a* KO cells, cellular levels of lysosomal glycosidases and lysozyme are decreased, and lysosomal pH is increased. Surprisingly, overexpression of Kil1 restores efficient killing in *phg1a* KO cells without correcting these lysosomal anomalies. Conversely, *kil1* KO cells are defective for killing, but their enzymatic content and lysosomal pH are indistinguishable from WT cells. The killing defect of *phg1a* KO cells can be accounted for by the observation that in these cells the stability and the cellular amount of Kil1 are markedly reduced. Since Kil1 is the only sulfotransferase characterized in Dictyostelium, an (unidentified) sulfated factor, defective in both *phg1a* and *kil1* KO cells, may play a key role in intracellular killing of *Klebsiella* bacteria. In addition, Phg1B plays a redundant role with Phg1A in controlling cellular amounts of Kil1 and intracellular killing. Finally, cellular levels of Kil1 are unaffected in *kil2* KO cells, and Kil1 overexpression does not correct the killing defect of *kil2* KO cells, suggesting that Kil2 plays a distinct role in intracellular killing.

## Introduction

*Dictyostelium discoideum* is a free-living amoeba found in the soil, where it feeds on bacteria. It has largely been used as a genetic system to study cellular mechanisms involved in phagocytosis and subsequent bacterial killing [1]. In this system, Phg1A was originally identified as a protein necessary for efficient adhesion, the first step of the phagocytic process [2]. Phg1A belongs to the TM9 family of proteins, defined by their 9 transmembrane domains and a high degree of conservation. The TM9 family comprises three members in *Dictyostelium* (Phg1A, B and C) and yeast (TMN1, 2 and 3), and four in *Drosophila* and human (TM9SF1 to 4). Remarkably, TM9 proteins also play an important role in adhesion in yeast [3] and *Drosophila* [4], and their overexpression in human metastatic melanoma cells conferred the pathogenic ability to engulf neighboring cells [5]. In a recent study, we showed that in *Dictyostelium*, Phg1A participates in cell adhesion by controlling the surface expression of SibA adhesion molecules [6].

Besides the role of Phg1A in adhesion, previous work has shown that *phg1a* knockout (KO) cells are unable to kill efficiently *Klebsiella* bacteria, and consequently to grow on a lawn of *Klebsiella* bacteria [7]. This could potentially be due to the fact that *phg1a* KO cells also fail to retain efficiently lysosomal enzymes in lysosomes [3]. This loss of intracellular enzymes may, in principle, account for the killing defect observed in *phg1a* mutant cells. In addition, Phg1A-depleted mammalian cells were shown to exhibit an abnormally high lysosomal pH [5]. Defective lysosomal acidification, if also observed in *Dictyostelium*, could also in principle affect intracellular killing. Further genetic analysis has yielded tools that can be used to test these hypotheses. In particular, the sulfotransferase Kil1 was identified as a protein whose overexpression restores efficient killing of *Klebsiella* in *phg1a* KO cells [7]. In addition, *kil1* KO cells fail to kill efficiently internalized *Klebsiella* bacteria [7]. Finally, Kil2, a P-type ATPase potentially transporting magnesium ions into phagosomes, is necessary for optimal activity of phagosomal proteolytic enzymes, and for efficient killing of *Klebsiella* bacteria [8].

In this study we characterized various mutant strains to define the functional relationships between Phg1A, Phg1B, Kil1 and Kil2, and their respective roles in intracellular killing of bacteria.

## Materials and Methods

### Strains and Media

*Dictyostelium discoideum* strains were grown at 21°C in HL5 medium [9]. All strains used in this study derived from the DH1–10 sub-clone of the *Dictyostelium* axenic strain DH1 [2], which is referred to as wild-type (WT) for simplicity. We have described previously the mutant strains *phg1a* KO [2], *phg1b* KO, *phg1a* KO overexpressing Phg1B [10], *phg1a* KO overexpressing Kil1, *kil1* KO [7] and *kil2* KO [8]. *Kil2* KO cells overexpressing Kil1 were obtained in this study by transfecting *kil2* KO cells with a Kil1 expression plasmid [7].

To assess growth of *Dictyostelium* on bacteria, *Klebsiella* bacteria were grown overnight at 37°C in LB. After centrifugation and resuspension in phosphate buffer (PB: 2 mM Na_2_HPO_4_, 14.7 mM KH_2_PO_4_, pH 6.5), the bacteria recovered from 30 ml of culture were plated on a PB-agar plate together with 30 *Dictyostelium* cells. Plates were scanned after 7 days of growth at 21°C to visualize growth of individual *Dictyostelium* clones. Note that in this assay the conditions in which the bacteria are grown (37°C, LB) are identical to the conditions used to grow bacteria prior to measuring killing of bacteria by *Dictyostelium* (see below).

### Activity of Lysosomal Enzymes

Secretion of lysosomal glycosidases was measured out as previously described [3]. Briefly, cells were grown for 3 days until they reached a density of 2–3×10^6^ cells/ml. Cells and medium were separated by centrifugation (1′500×*g*, 2 min), and the presence of glycosidase activity (N-acetyl β-glucosaminidase and α-mannosidase) assessed using specific chromogenic substrate (p-nitrophenyl n-acetyl beta-D glucosamide and p-nitrophenyl alpha-D-mannopyranoside, respectively). Accumulation of para-nitrophenol upon glycolysis was measured by spectrophotometry (405 nm).

We measured lysozyme activity essentially as described [11]. Briefly, a 2 mm-thick layer of agarose (0.9%) containing 50 mM sodium-acetate pH 4.5 and 0.5 mg/ml lyophilized cell wall from *Micrococcus lysodeikticus* (M3770 Sigma-Aldrich), was poured in sterile Petri dishes and several 4 mm-diameter holes were created. *Dictyostelium* cells were grown in suspension (10^6^ cells/ml), 100 ml of cell suspension was centrifuged (1500×*g*, 2 min), then washed with 20 ml of phosphate buffer (PB). Each pellet was resuspended in 10 times its volume of 10% acetic acid containing proteases inhibitors (5 mg/ml iodoacetamide, 47 µM leupeptin, 1.5 µM aprotinin and 100 µM phenylmethylsulfonylfluoride) and was rotated on a wheel at 4°C overnight. Additional protein extraction was performed by sonication of the extract for 15 min in a sonicator bath. Finally, the cell extract was centrifuged in an airfuge (150′000×*g*, 4°C, 1 h) and the supernatant was collected. Defined volumes of supernatant (2–20 µl) (completed to 20 µl), were deposited in holes on an Agarose-*Micrococcus* plate, and the plate incubated at 37°C for 24 h. Activity of lysozyme created a clear halo around the holes. The relative lysozyme activity in mutant cells was assessed by comparing the halo diameter with that created by applying various dilutions of WT extracts.

### Kinetics of Endosomal Acidification

Acidification of endosomal compartments was measured as described previously [12]. Briefly, 2.2×10^6^ cells were allowed to endocytose HL5 pH 7.4 containing 250 µg/ml Oregon Green 488-coupled dextran (Invitrogen) and 30 µg/ml Alexa 647-coupled dextran (Invitrogen). After 20 min of endocytosis, cells were washed in 1 ml HL5 pH 7.4 and resuspended in 2.2 ml of HL5 pH 7.4. At each time point, 200 µl of cells were collected and immediately analyzed by flow cytometry. A calibration curve was obtained by measuring the fluorescence of cells when a defined endosomal pH (from 3.5 to 6) was imposed by the addition of NH_4_Cl and sodium azide.

### Phagosomal Proteolysis

Proteolysis of BSA coupled to ingested beads was followed essentially as described [13]. Briefly, 2.5×10^6^ cells were washed with PB and transferred in a 2 ml eppendorf tube in 1.5 ml PB. Cells were allowed to engulf 3 µm carboxylated silica beads (Kisker Biotech) (1 bead for 10 cells) coupled to Alexa Fluor 594 succinimidyl ester (Molecular Probes) and to BSA labeled with DQgreen at a self-quenching concentration (Molecular Probes). Tubes were rotated on a wheel for 15 min to allow phagocytosis. Then at each time point (0, 15, 30, 60 and 120 min), 200 µl of the cell suspension were collected and analyzed by flow cytometry. Flow cytometry allowed to discriminate free beads, cells (lower left corner) and cells containing internalized beads (R region). To evaluate proteolysis of BSA, the median intensity of the DQgreen fluorescent marker was measured in cells containing phagocytosed beads (R region).

### Amount and Stability of Cellular Kil1

To evaluate the amount of Kil1 in each strain, 2×10^6^ cells were pelleted and resuspended in 80 µl of sample buffer (0.103 g/ml sucrose, 5×10 mM Tris, pH 6.8, 5×10 mM EDTA, 0.5 mg/ml bromophenol blue, 2% SDS). Twenty µl of each sample was migrated on a 9% acrylamide gel, and transferred to nitrocellulose using a semi-dry transfer system (Invitrogen, Carlsbad, CA). The membrane was incubated overnight in PBS containing 0.1% Tween 20 and 7% milk, then incubated successively with a rabbit anti-Kil1 serum [7], and a horseradish peroxidase-coupled goat anti-rabbit IgG. The signal was revealed by enhanced chemiluminescence and quantified using a Biorad Chemidoc system.

To determine the turnover of Kil1, 5×10^6^ cells were incubated in HL5 containing 2 mM cycloheximide. Aliquots of 1.5×10^6^ cells were collected after 0, 2 and 4 h and resuspended in 20 µl of SB. The amount of Kil1 in each sample was determined by Western blot as described above.

### Killing of Klebsiella Bacteria by Dictyostelium

The ability of *Dictyostelium* strains to kill internalized bacteria was tested as described previously [7]. Briefly, 2×10^6^ *Dictyostelium* cells were collected, washed in PB, resuspended in 500 µl of PB containing *Klebsiella aerogenes* (2×10^4^/ml) grown overnight at 37°C in LB, and incubated at 21°C. After 0, 1, 2 and 4 h, 10 µl-aliquots were collected, mixed to 100 µl-aliquots of PBS containing 0.1% Triton X100 to kill *Dictyostelium* cells, and plated on LB-Agar plates. Live bacterial clones were counted after an overnight incubation at 37°C.

## Results

### Lysosomal Physiology is Affected in *phg1a* Mutant Cells

**Figure 1:**
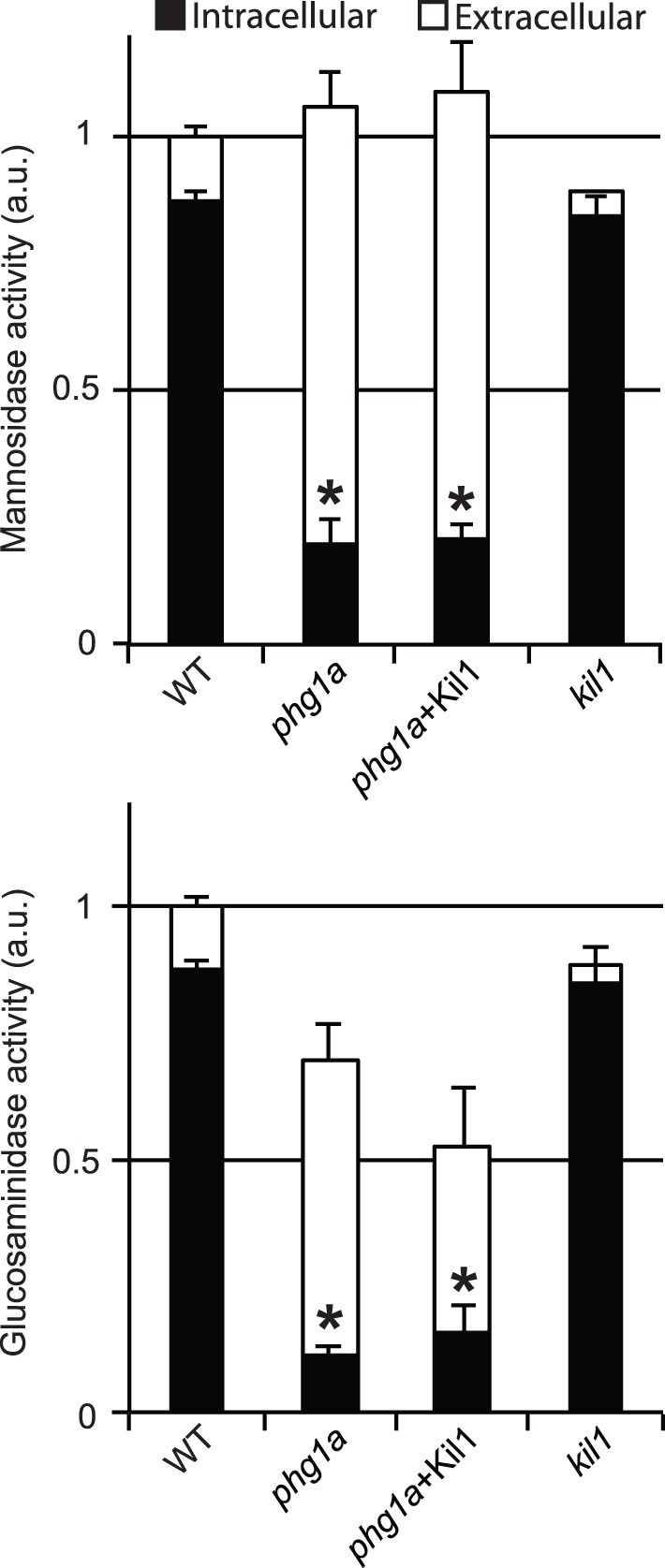
Mislocalization of lysosomal glycosidases in *phg1a* KO is not complemented by Kil1 overexpression. (A) The intracellular activity of lysosomal alpha-mannosidase was assessed in cellular pellets (intracellular) and in the cell culture medium (extracellular) for WT, *phg1a* KO, *phg1a*+Kil1 and *kil1* KO cells. The total activity (intracellular+extracellular) was similar in every strain. However the amount of intracellular mannosidase was significantly reduced in *phg1a* KO and *phg1a* KO overexpressing Kil1 compared to WT cells. (B) Similar results were obtained when another lysosomal glycosidase (N-acetyl glucosaminidase) was tested. The average and S.E.M. of 5 experiments are presented. * : significantly different from WT (Student t-test; p<0.01).

**Figure 2:**
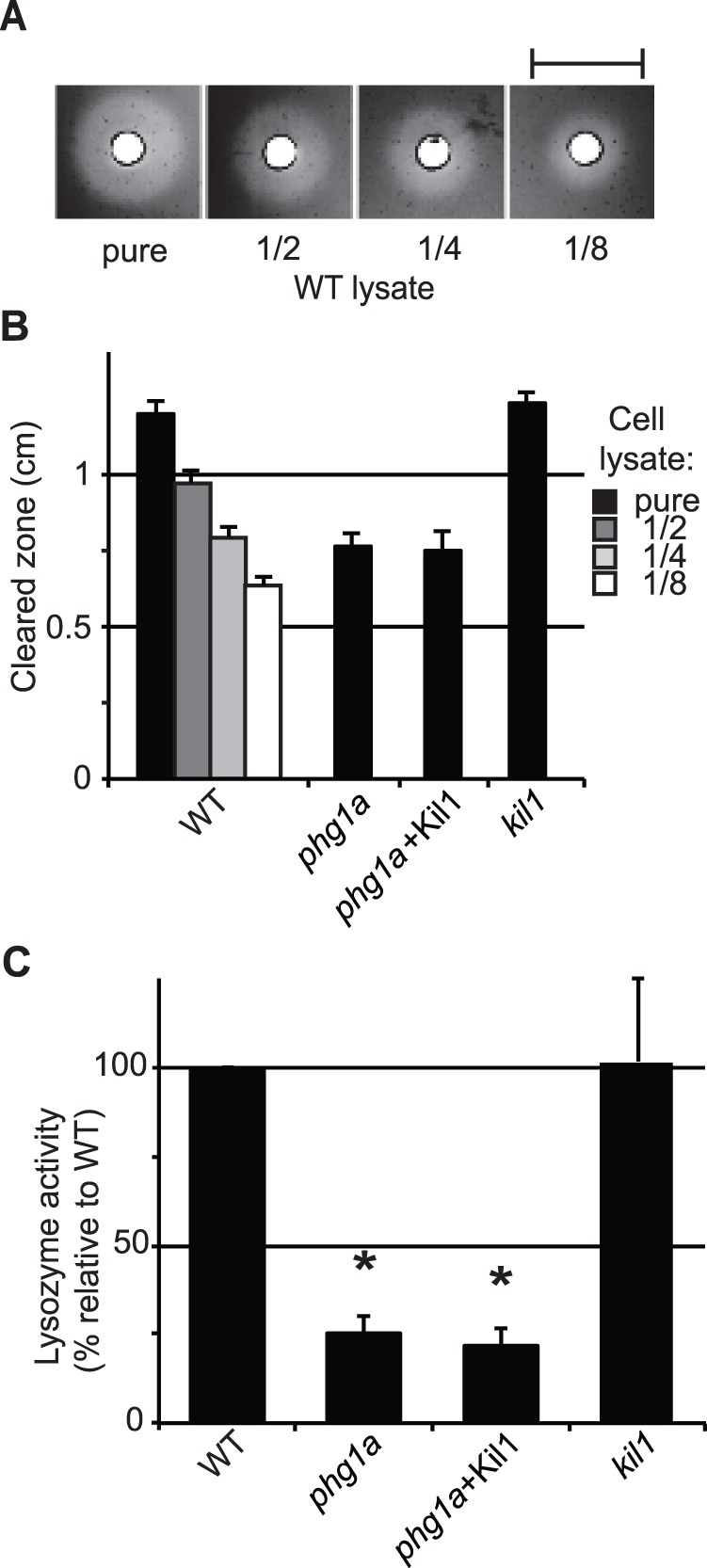
Cellular lysozyme activity is decreased in *phg1a* KO cells. A) Lysate from WT cells were deposited on an agarose plate containing cell wall extracts of *M. lysodeikticus*. Lysozyme present in the lysate digests the bacterial cell wall, forming a cleared zone on the plate, the size of which decreased when the cellular lysate was diluted. Scale bar: 1cm. B) Average diameter of cleared zones for different concentrations of WT lysates provides a scale to which undiluted mutant cell lysates can be compared (n≥4) C) Lysozyme relative activity was deduced from results presented in B, after normalization. * : significantly different from WT (Student t-test; p<0.01).

Previous work has shown that *phg1a* KO cells present defects in the sorting of lysosomal glycosidases and of cathepsin, leading them to secrete these enzymes and to retain intra-cellularly a reduced amount ([3] and Fig. 1). Here we assessed in addition the cellular content of lysozyme, an enzyme involved in digestion of bacterial cell wall and that is thought to participate in bacterial killing [14]. This enzyme hydrolyses the 1,4-β-linkages between N-acetylmuramic acid and N-acetyl-D-glucosamine residues of peptidoglycans, a key component of bacterial wall [15]. The *Dictyostelium* genome exhibits 11 lysozyme genes, the main one (AlyA) representing more than half of the total cellular activity [16]. To measure cellular lysozyme activity in *Dictyostelium*, we used a test similar to the previously described halo assay [11]. For this, cellular extracts were deposited on an agarose plate containing cell walls of *Micrococcus lysodeikticus*. Lysozyme digests the bacterial cell wall, and forms a clear halo, the size of which reveals the level of lysozyme activity (Fig. 2A). This assay showed that the amount of lysozyme is strongly reduced in *phg1a* mutant cells compared to wild-type (WT) cells (Fig. 2B and C).

**Figure 3:**
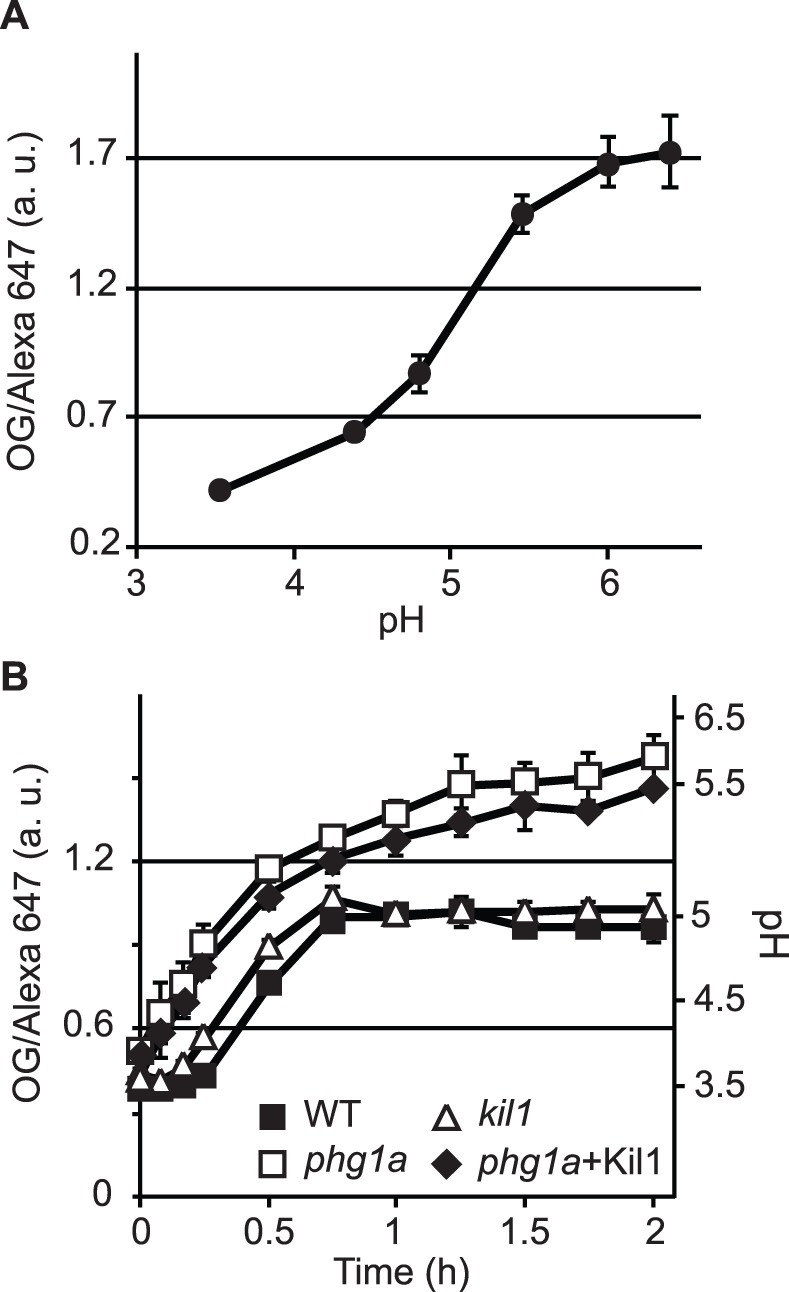
Endosomal acidification defects in *phg1a* KO cells are not corrected by overexpression of Kil1. In order to measure the acidification of endosomal compartments, *Dictyostelium* cells were allowed to engulf fluid phase containing two dextran-coupled fluorescent markers, one sensitive to pH (Oregon Green 488), the other one not (Alexa 647). A) The calibration curve indicates the OG/Alexa 647 ratio obtained by imposing different endosomal pH (3.5 to 6.5). B) Kinetics of endosomal acidification in WT or mutant cells. The left axis indicates the OG/Alexa 647 ratio, the right axis the corresponding pH values. The average and S.E.M. of at least 3 independent experiments are presented.

A study in mammalian cells has indicated that acidification of endosomal compartments is defective in cells where *PHG1A* has been silenced [5]. This led us to measure the endosomal pH in *Dictyostelium* cells, by following the pH of the compartments encountered by an internalized pH-sensitive fluorescent fluid phase marker [12]. A calibration curve can be obtained by imposing known pH values in endosomes (Fig. 3A). In WT cells, fluid phase reaches rapidly very acidic lysosomes, and is then gradually transferred after 20–30 min to late, less acidic post-lysosomes (Fig. 3B). Acidification of both early and late endosomal compartments was significantly less pronounced in *phg1a* KO cells than in WT cells (Fig. 3B).

These observations indicate that Phg1A is necessary to ensure intracellular accumulation of lysosomal glycosidases and lysozyme, as well as proper acidification of lysosomes. This result suggested that the inability of *phg1a* mutant cells to kill efficiently *Klebsiella* bacteria may be the result of a defective lysosomal acidification, or a loss of lysosomal enzymes involved in bacterial killing, or a combination of both defects.

### Kil1 Overexpression in *phg1a* KO Cells Restores Killing, but not Lysosomal Physiology

In order to characterize further the cause of the killing defect observed in *phg1a* mutant cells, we made use of a *phg1a* KO strain overexpressing Kil1, which was previously shown to kill intracellular bacteria as efficiently as WT cells [7]. To our surprise, we observed that *phg1a* cells overexpressing Kil1 exhibit the same reduced level of intracellular enzymatic activity as *phg1a* KO cells (Fig. 1). Similarly, lysozyme activity in *phg1a* KO remained very low even upon overexpression of Kil1 (Fig. 2C). Finally, the lysosomal pH remained also abnormally high in *phg1a* cells overexpressing Kil1 (Fig. 3). Together, these results indicate that overexpression of Kil1 restores a normal killing activity in *phg1a* KO cells, without restoring a normal lysosomal physiology. These observations suggest that the partial loss of lysosomal enzymes and the elevation of the lysosomal pH in *phg1a* mutant cells are not key elements accounting for a decreased bacterial killing activity.

### Loss of Kil1 Impairs Bacterial Killing, but not Lysosomal Physiology

To characterize the role of Kil1 in intracellular killing, we studied the physiology of lysosomal compartments in *kil1* KO cells. *Kil1* KO cells contained lysosomal glycosidase as well as lysozyme levels similar to those found in WT cells (Fig. 1 and 2). In addition, acidification of endocytic compartments was indistinguishable in *kil1* KO and in WT cells (Fig. 3). These observations indicate that Kil1 participates in killing without being directly involved in the control of lysosomal physiology.

**Figure 4:**
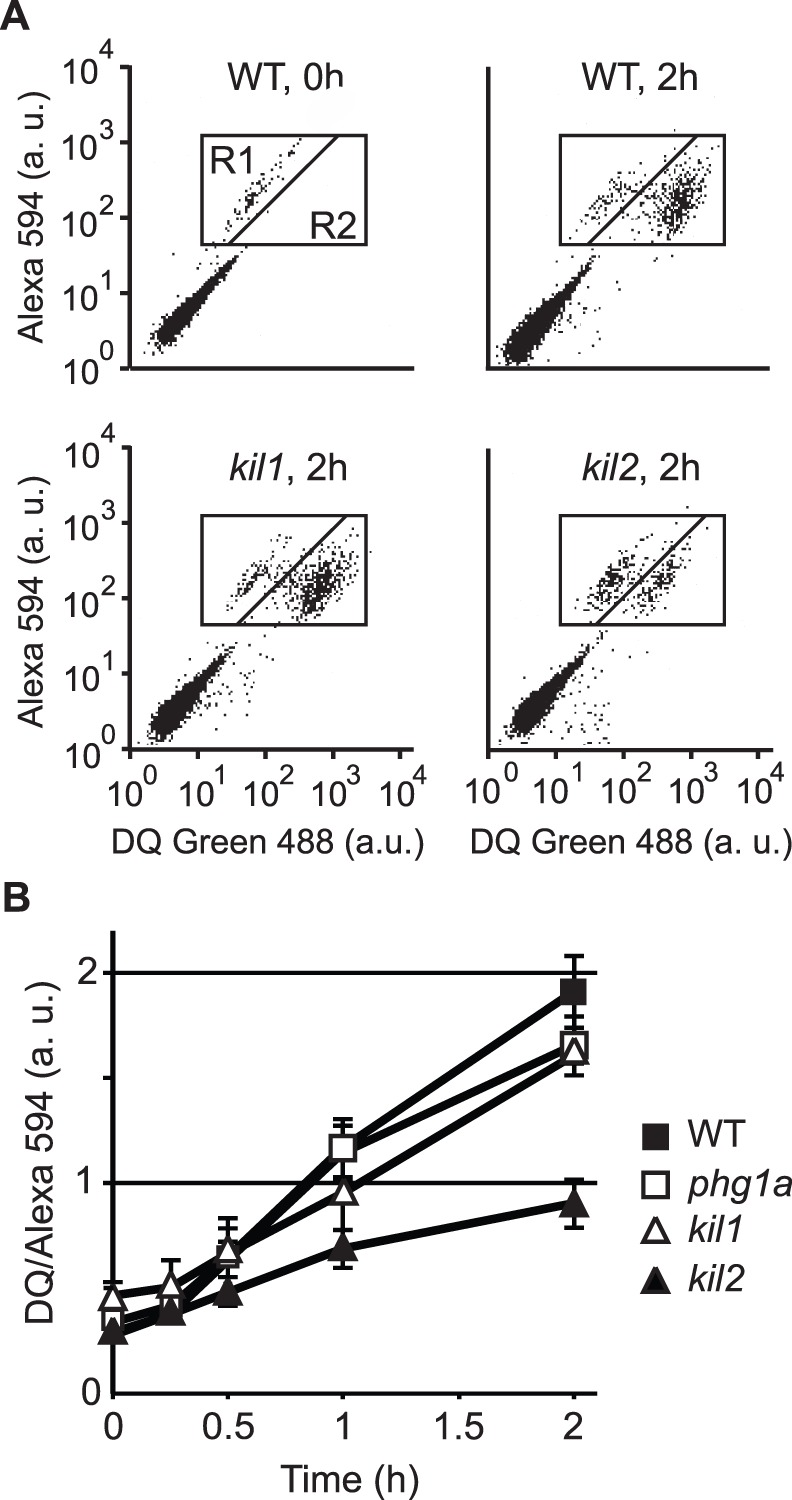
Phagosomal proteolysis is not defective in *kil1* KO cells. Cells were allowed to engulf silica beads coupled to Alexa Fluor 594 and to BSA labeled with DQgreen at a self-quenching concentration. Proteolysis of BSA in phagosomal compartments released DQgreen fluorescence, which was measured by flow cytometry in the R1+R2 region (cells containing fluorescent beads). A) WT cells having just phagocytosed beads (WT, 0 h) showed a low fluorescence (R1 window). After 2 h of incubation (WT, 2 h), the increase in DQgreen fluorescence revealed intra-phagosomal proteolytic activity. A similar pattern was observed in *kil1* KO cells (*kil1*, 2 h), but not in *kil2* KO cells (*kil2*, 2 h) where phagosomal proteolytic activity is reduced [8]. B) Cell-associated DQ green fluorescence was measured in the R1+R2 region after various times of incubation. The average and S.E.M. of 4 independent experiments are presented.

The killing defect of *kil1* KO cells could be due to the mislocalization or loss of a protease not detected in our assays. To test this hypothesis, we measured in living cells the protease activity in phagosomes (Fig. 4). For this, we allowed cells to phagocytose silica beads coated with BSA onto which two fluorescent probes were attached. The fluorescence of one of the probes (DQ green) is quenched when attached to the beads, but increases when it is released following proteolysis of BSA. The cells were analyzed by flow cytometry to determine the level of fluorescence of internalized beads as a function of time, which provides an estimate of the efficiency of intra-phagosomal proteolysis (Fig. 4A). The resulting curves showed that *kil1* KO cells digested BSA as efficiently as WT cells (Fig. 4B and C). As a control we observed that *kil2* KO cells, for which a defective proteolytic activity has previously been reported [8], did not proteolyse BSA efficiently (Fig. 4B and C).

In summary, although *kil1* KO cells kill *Klebsiella* inefficiently, none of the parameters tested revealed an alteration of their lysosomal physiology.

### Phg1A Controls Cellular Amounts of Kil1

**Figure 5:**
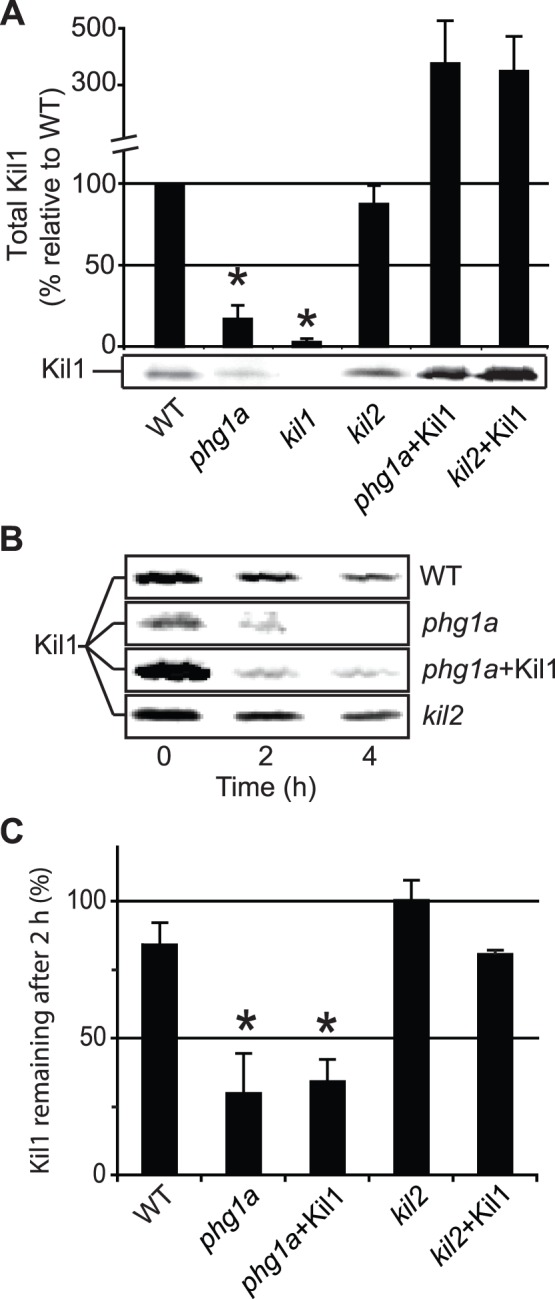
The stability of Kil1 is impaired in *phg1a* KO. A) The amount of Kil1 protein was revealed by Western blot in lysates from WT and mutant cells and quantified in at least 3 independent experiments. A significant decrease of the cellular amount of Kil1 was observed in *phg1a* mutant cells compared to WT cells. B) The stability of the Kil1 protein was monitored by incubating cells in the presence of cycloheximide, an inhibitor of protein synthesis, for 4 h. The amount of Kil1 was assessed by Western blot after 0, 2 and 4 h. C) The amount of Kil1 protein remaining after 2 h was expressed as a percentage of the amount measured at time 0. The average and S.E.M. of 4 independent experiments are presented. *: significantly different from WT (Student t-test; p<0.05).

In a recent study, we have shown that Phg1A controls the cellular amounts of SibA, a protein involved in cellular adhesion [6]. In *phg1a* KO cells, SibA is produced less efficiently and degraded more readily than in WT cells [6]. Phg1A may similarly affect other cellular functions by controlling the amounts of other membrane proteins. In order to test this hypothesis, we assessed the amount of Kil1 protein present in *phg1a* mutant cells. The Kil1 protein appeared largely depleted in *phg1a* KO cells compared to WT cells (Fig. 5A). We then tested the stability of the Kil1 protein in WT cells, *phg1a* KO cells, and *phg1a* KO cells overexpressing Kil1. For this, cells were incubated for various times in the presence of cycloheximide, an inhibitor of protein synthesis, and the amount of Kil1 was assessed by Western blot (Fig. 5B). To allow a more quantitative analysis, we measured the relative amount of cellular Kil1 protein remaining after 2 h of chase in four independent experiments. In WT cells, the Kil1 protein was relatively stable (14% of the protein degraded after 2 h of chase), while in *phg1a* KO cells, 69% of the Kil1 protein was degraded after a 2 h chase. Similarly in *phg1a* KO cells overexpressing Kil1, 66% of Kil1 was degraded after 2 h of chase. These results demonstrate that the Kil1 protein is less stable in *phg1a* KO cells than in WT cells, and this accounts for the reduced amount of Kil1 protein in *phg1a* KO cells. Since a loss of Kil1 is sufficient to cause a killing defect, lack of Kil1 could be sufficient to cause the killing defect of *phg1a* mutant.

### Phg1B Controls Cellular Levels of Kil1 and Intracellular Killing

**Figure 6:**
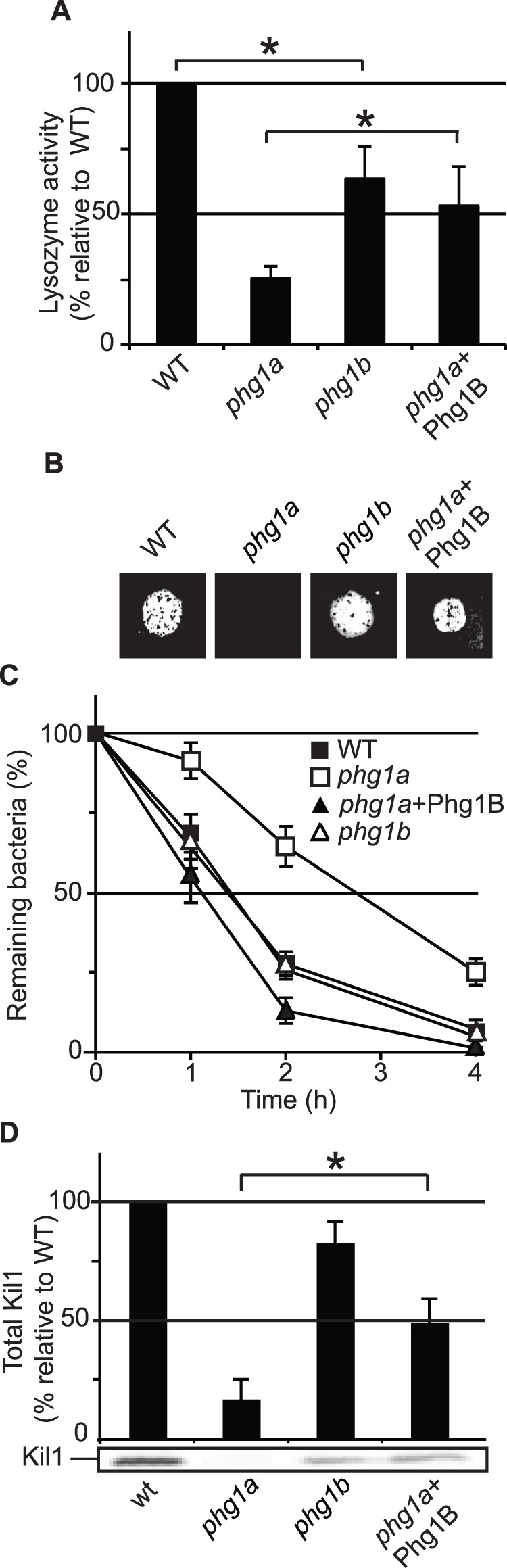
Phg1A and Phg1B play redundant roles in controlling intracellular killing and Kil1 stability. A) The amount of cell-associated lysozyme activity was measured as described in figure 2. As observed previously for lysosomal glycosidases [3], the cellular activity of lysozyme was reduced in *phg1B* KO cells compared to WT cells, and enhanced by Phg1B overexpression in *phg1A* KO cells. B) WT or mutant cells were grown on a lawn of *Klebsiella* bacteria. Loss of Phg1B was not sufficient to inhibit growth on *Klebsiella*, but overexpression of Phg1B in *phg1A* KO cells restored growth on bacteria. C) The ability of WT and mutant *Dictyostelium* cells to kill *Klebsiella* bacteria was measured, revealing that Phg1B overexpression restores efficient killing in *phg1a* KO cells (average and S.E.M. of 4 experiments). D) The total cellular amount of Kil1 was measured in WT and mutant cells. The intracellular level of Kil1 was increased in *phg1a* KO cells by overexpressing Phg1B. Average of at least 4 experiments. *: significantly different (Student t-test; p<0.05).

Phg1A and Phg1B have been shown previously to exhibit partially redundant functions. Specifically, overexpression of Phg1B has been shown to restore partially sorting of lysosomal glycosidases in *phg1a* KO cells [3]. Similarly, in *phg1b* KO cells, the intracellular level of lysozyme was reduced, and overexpression of Phg1b in *phg1a* KO cells increased the intracellular level of lysozyme (Fig. 6A). To assess the putative role of Phg1B in intracellular killing, we tested growth on bacteria of *phg1a* KO and *phg1b* KO cells as well as *phg1a* KO cells overexpressing Phg1B. *Phg1b* KO cells were still able to grow on *Klebsiella*, and to kill them efficiently (Fig. 6B and C). Overexpression of Phg1B in *phg1a* KO restored the ability of *phg1a* KO cells to kill *Klebsiella* and to grow upon them (Fig. 6B and C). Interestingly, loss of Phg1b caused a slight decrease of cellular Kil1, while overexpression of Phg1b in *phg1a* KO cells significantly increased the cellular amount of Kil1 (Fig. 6D).

These results indicate that Phg1B and Phg1A play largely redundant roles in controlling lysosomal physiology as well as the intracellular levels of Kil1, although Phg1A appears to play a quantitatively more important role. In light of the results presented above, we suggest that the main role of Phg1B in intracellular killing, like that of Phg1A, is to control the cellular level of Kil1.

### Kil2 Plays a Kil1-independent Role in Killing

**Figure 7:**
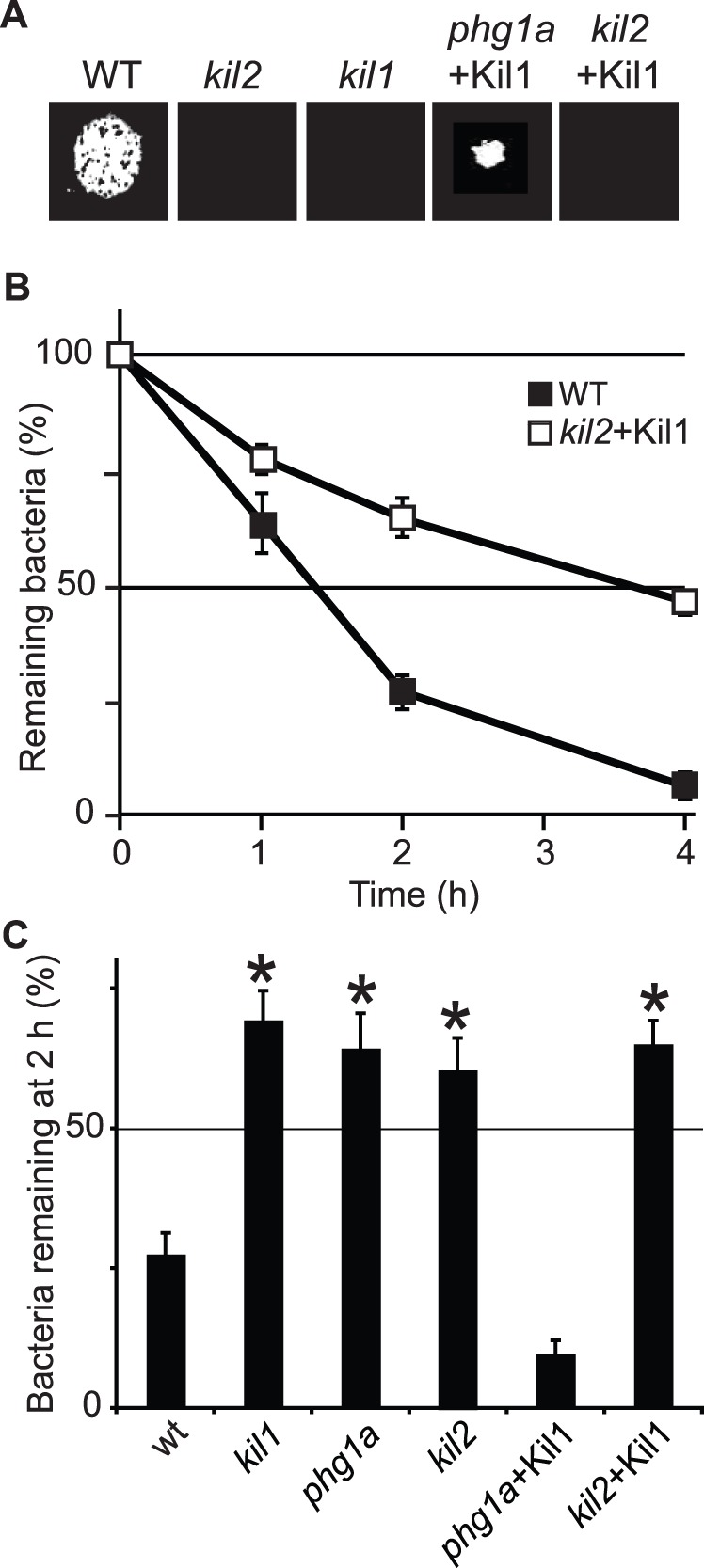
Kil1 overexpression restores efficient killing in *phg1a* but not in *kil2* KO cells. A) The ability of WT and mutant cells to grow on *Klebsiella* was tested as described in Figure 6B. B,C) The ability of WT and mutant cells to kill *Klebsiella* was tested and quantified as described in Figure 6C. Overexpression of Kil1 did not restore the ability of *kil2* KO cells to grow on bacteria or to kill them efficiently. The average and S.E.M. of 6 experiments are presented. * : significantly different from WT (Student t-test; p<0.01).

In order to unmask possible functional links between the Kil1 and Kil2, we assessed the effect of Kil1 overexpression on the phenotype of *kil2* KO cells. Kil1 overexpression did not restore the ability of *kil2* KO cells to kill bacteria and to feed upon them (Fig. 7). Conversely, in *kil2* mutant cells neither the cellular levels, nor the stability of Kil1 were affected compared to WT cells (Fig. 5). These results suggest that the roles of Kil1 and Kil2 in intracellular killing are functionally distinct.

## Discussion

In this work, we characterized the phenotypes of selected *Dictyostelium* strains, in order to identify the key factors involved in intracellular bacterial killing. Our results show that loss of Phg1A causes an alteration of lysosomal physiology characterized by an elevation of pH and a decrease of the intracellular levels of lysosomal enzymes and lysozyme. However, contrary to our expectations, these features were not essential to account for defective killing in *phg1a* KO cells, since efficient killing was restored in *phg1a* KO cells by overexpression of Kil1 without correcting lysosomal anomalies. In agreement with this, loss of Kil1 was sufficient to impair intracellular bacterial killing, without altering lysosomal pH or enzymatic levels. We further observed that Kil1 stability is decreased in *phg1a* KO cells, resulting in very low levels of Kil1 protein in these cells. Together these observations suggest that the killing defect of *phg1a* KO cells is mainly, and maybe fully, due to the loss of Kil1 in these cells.

While our observations stress the critical role of Kil1 in intracellular killing of bacteria, they fail to provide positive evidence accounting for this role. Kil1 is the only characterized sulfotransferase in *Dictyostelium*. Although other putative sulfotransferases can be identified in the genome (e.g. gtr1), no sulfated glycoproteins were detected in *kil1* KO cells with an antibody specific for proteins carrying a mannose-6-sulphate-containing epitope [7]. In mammals, sulfated glycoproteins can be involved in a wide range of functions, such as cell adhesion, cell signaling, or intracellular transport and are essential for development [17]. It is striking to observe that in *Dictyostelium,* the apparently complete loss of sulfation in *kil1* KO cells results in a relatively subtle phenotypic alteration. Indeed we were unable to detect any other defect than a killing defect in *kil1* KO cells, and they were notably undistinguishable from WT cells for lysosomal physiology (this study), phagocytosis [7] or cell growth. Our results suggest the existence of a sulfated factor playing a key role in the intracellular killing of *Klebsiella* bacteria, which remains to be identified.

**Figure 8:**
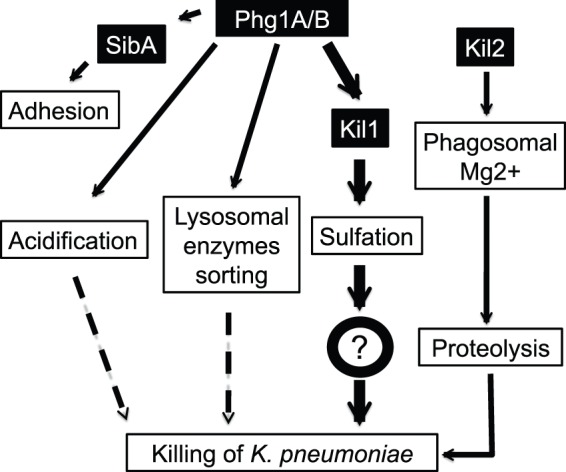
Role of Phg1 proteins in cell adhesion and intracellular killing, a model. According to the results of this study, Phg1A and B proteins regulate lysosomal physiology (pH, enzymes), but this has a limited impact on intracellular killing of bacteria. The primary role of Phg1 proteins in intracellular killing is to control the cellular amount of Kil1. Kil1 is necessary for efficient intracellular killing, although it precise role in this process remains to be established. Kil2 independently controls intraphagosomal magnesium levels and protease activity. The effect of Phg1 proteins on cellular adhesion is due to their role in controlling surface expression of SibA.

The function of two other gene products was evaluated in this study: Phg1B can control intracellular killing, as evidenced by the fact that Phg1B overexpression restores efficient killing in *phg1a* KO cells. It seems likely that Phg1B participates in killing, like Phg1A, mainly by controlling the cellular levels of Kil1, since overexpression of Phg1B increases cellular Kil1 levels in *phg1a* KO cells. Finally Kil2 apparently belongs to an entirely distinct functional class, as evidenced by the fact that first, its loss does not affect the stability of Kil1, second overexpression of Kil1 in *kil2* KO cells does not restore efficient killing in these cells, and third loss of Kil1 does not affect intra-phagosomal proteolysis, while loss of Kil2 does. These results are summarized in Figure 8, which proposes an overview of the functional relationships between these different gene products.
